# Fanconi Syndrome in an Adult With Chronic Alcohol Use Disorder: A Rare Etiology

**DOI:** 10.7759/cureus.28205

**Published:** 2022-08-20

**Authors:** Shahzad Safdar, Shazmah Shahrukh, Ameerdad Khan, Zoha Shahid

**Affiliations:** 1 Nephrology, The Christ Hospital, Cincinnati, USA; 2 Internal Medicine, Dow University of Health Sciences (DUHS) Civil Hospital Karachi, Karachi, PAK; 3 Internal Medicine, Shifa International Hospital Islamabad, Islamabad, PAK; 4 Internal Medicine, Liaquat National Hospital and Medical College, Karachi, PAK

**Keywords:** hypouricemia, acute kidney injury, renal tubular defect, chronic alcoholism, fanconi syndrome, proximal tubular defect

## Abstract

Fanconi syndrome is described as a defect in the proximal tubular reabsorption of glucose, amino acids, uric acid, phosphate, and bicarbonate, falling under type 2 renal tubular acidosis (RTA). Some common causes include drugs, heavy metals, infections, and genetics (particularly mitochondrial disorders).

We present a case of a 33-year-old Caucasian female with chronic alcohol use disorder. She was treated for acute kidney injury (AKI) but had persistent hypophosphatemia, hypokalemia, hypouricemia, low bicarbonate, along with glycosuria consistent with Fanconi syndrome. An exhaustive workup ruled out the most common causes. Alcohol abstinence proved to correct the underlying abnormality.

Alcohol is a mitochondrial toxin, and its role in the pathophysiology of Fanconi syndrome is under investigation. Early diagnosis of Fanconi is imperative to avoid complications such as rickets and osteomalacia. Therefore, testing for markers of alcohol abuse should be considered when determining the etiology of Fanconi syndrome.

Alcohol use disorder is a common disorder, with more than 3 million cases annually in the US alone. Clinicians should have a high index of suspicion for Fanconi syndrome in a patient with similar anomalous labs considering the high prevalence of alcohol use disorder. More research regarding this topic is warranted.

## Introduction

Fanconi syndrome is described as a defect in the proximal tubular reabsorption of glucose, amino acids, uric acid, phosphate, and bicarbonate, falling under type 2 renal tubular acidosis (RTA) [1]. RTA is characterized by an inability to maintain normal acid-base homeostasis. Treatment of the syndrome is targeted at treating the underlying disease pathology and fluid repletion. Acquired forms are more prevalent in the adult population, and known etiologies include drugs such as tenofovir [2] and cisplatin, lead poisoning, and autoimmune diseases like Sjogren’s syndrome and primary biliary cholangitis [3]. 

Alcohol use disorder (AUD) is one of the most prevalent psychiatric disorders globally, with a 12-month prevalence of 6.4% [4]. Chronic alcohol consumption is a well-known risk factor for tissue injury. Studies conducted primarily in other organs and tissues suggest several possible mechanisms by which alcohol may promote kidney dysfunction, including oxidative stress and mitochondrial dysfunction [5].

## Case presentation

A 33-year-old Caucasian female presented to the ED with complaints of intractable non-bloody, non-bilious vomiting and generalized fatigue for three days. She reported recurrent episodes of vomiting lasting 2-3 days every two months that were relieved by ondansetron. She also reported longstanding numbness and paresthesia in her feet for the past year.

There was no history of fever, dizziness, abdominal pain, diarrhea, or shortness of breath. She denied smoking or illicit drug use but admitted to consuming large quantities of alcohol in the past. Past medical history was significant for hypertension (HTN), hyperlipidemia, and rheumatoid arthritis. Home medications included amlodipine and rosuvastatin for hypertension and hyperlipidemia, respectively, along with treatment for seronegative rheumatoid arthritis with Humira (adalimumab) in the past, which was discontinued two years ago. She had a previous hospital admission for alcohol intoxication. Her labs revealed elevated ethanol levels of 1839 mg/dl warranting a liver biopsy that showed steatosis in accordance with her heavy alcohol use. There was no significant past surgical history, recent steroid, nonsteroidal anti-inflammatory drugs (NSAIDs), or antibiotic use.

On arrival, her vitals were normal. She was mildly ill-appearing but in no acute distress, had no resting tremors, and had a normal abdominal exam. No other significant examination findings were present. Lab work (Table 1) revealed elevated creatinine of 4.05 (baseline 0.85), blood urea nitrogen (BUN) of 48, and anion gap of 28. She was admitted for acute kidney injury (AKI) secondary to volume depletion and treated with IV fluids, electrolytes, and supportive care. 

**Table 1:** Lab values during initial presentation, after treatment of AKI, and after alcohol cessation. AKI: Acute kidney injury; BUN: Blood urea nitrogen; eGFR CKD EPI: Glomerular filtration rate chronic kidney disease epidemiology collaboration.

Labs	Initial presentation	After treatment of AKI	Four months of alcohol cessation	Normal range
Sodium	128	135	140	138-142 mmol/l
Potassium	2.9	3.3	3.8	3.5-5.0 mmol/l
Chloride	90	107	104	95-105 mmol/l
Bicarbonate	10	18	27	23-28 mEq/l
Anion Gap	28	10	9	8-12
BUN	48	17	13	6-18 mg/dl
Creatinine	4.05	1.85	0.85	0.6-1.2 mg/dl
Glucose	156	97	96	70-100 mg/dl
eGFR CKD EPI	16	31	93	90-120 ml/min/1.73m2
Calcium	10.5	8.9	10.5	8.6-10.3 mg/dL
Phosphorus	9.0	1.5	4.2	2.8-4.5 mg/dL
Albumin			4.1	3.4-5.4 g/dL
BUN/Creatinine ratio		9	15	10-20
Magnesium	2.7			1.3-2.1 mEq/L
Uric acid		<0.3		2.6-6.0 mg/dl

However, despite treatment, her lab results revealed hypophosphatemia (1.5), hypouricemia (<0.28 mg/dl; normal range 3.5-7 mg/dl), non-anion gap hyperchloremic metabolic acidosis, and hypokalemia. Urine chemistries were significant for proteinuria 1.6g/24hrs (normal <0.3g/24hrs), phosphaturia 12.4 mg/dl (normal 2.5-4.6 mg/dl), hyperuricosuria 32 mg/dl (normal 2.7-5.4 mg/dl) and low urinary sodium <20 mmol/l (lab values in Tables 1-2). On review of her previous lab work, similar results indicated a chronic underlying pathology. Glycosuria with euglycemia (urine glucose 500 mg/dl with normal serum glucose), hypokalemia (2.6; 3.3; 3.4) with high urine potassium, intermittently low bicarbonate (21; 19) were present on different occasions. Fractional excretion of phosphorus was 15% (above 5% indicates renal phosphate wasting) after treatment of her AKI.

**Table 2:** Urine chemistry. AKI: Acute kidney injury.

Urine chemistries	Initial presentation	After treatment of AKI	Normal ranges
Glucose	100		0-0.8 mmol/L
Protein	>300	1600	0-300 mg/24hrs
Ketones	40		<0.6
Phosphate		12.4	3.0-4.5 mg/dL
Sodium		<20	40-220 mEq/day

Probable underlying causes such as multiple myeloma, genetic diseases, fibromuscular dysplasia, metal poisoning, and Wilson's disease were ruled out with appropriate tests (Table 3). In addition, a kidney biopsy was done after AKI to reveal any underlying pathology and showed acute tubular injury, along with 1+ IgA mesangial deposition by immunofluorescence; no immune complexes were found on electron microscopy.

**Table 3:** Diagnostic studies performed. ANA: Antinuclear antibodies; ANCA: Anti-neutrophil cytoplasmic antibody; CCP: Cyclic citrullinated peptide; RF: Rheumatoid factor; HLA: Human leukocyte antigen; TTG: Tissue transglutaminase; HCV: Hepatitis C virus; HBV: Hepatitis B virus; HAV: Hepatitis A virus; SPEP: Serum protein electrophoresis; ALT: Alanine aminotransferase; AST: Aspartate Aminotransferase; LDL: Low-density lipoprotein; ACTH: Adrenocorticotropic hormone; ACE: Angiotensin-converting enzyme; TSH: Thyroid-stimulating hormone; SPECT: Single-photon emission computerized tomography; CT: Computed tomography; PLA2R: Phospholipase A2; TEE: Transesophageal echocardiogram; U/S: Ultrasound; EMG: Electromyography.

Test	Results	Test	Results
Autoimmune profile including ANA, ANCA, CCP, HLA, RF	Negative	Infectious workup for HIV, HCV, HBV and hAV, Syphilis	Negative
Celiac panel/TTG, IgA	Negative	Alpha actin	Negative
Complement C3 and C4	Negative	Serum and urine SPEP	Negative
1-25 Vitamin D	Negative	Blood cultures	Negative
ALT/AST	ALT: 45, AST: 71	Urine metal screen	Negative
LDL	Negative	Serum zinc	Negative
Cortisol/ACTH	Negative	Serum copper	Negative
Renin/Aldosterone	Negative	Serum B1	Negative
ACE	Negative	Serum B12	Negative
Thyroid panel (TSH, free T4, FREE T3)	Negative	Renal artery duplex	Negative
Free kappa lambda	Negative	EMG	Negative
Ceruloplasmin, copper stain	Negative	Nerve biopsy	Negative
SPECT scan	Negative	Renal ultrasound	Negative
TEE	EF 59-63% normal	Kidney biopsy (with congo red staining)	Negative 1+ IgA mesangial deposition
CT angiogram	Negative	Renasight genetic panel	Negative
PLA2R	Negative	IgA	Mildly high 335

The patient was discharged six days after hospitalization with improvement in BUN at 17 and creatine at 1.85 with advice to limit alcohol intake. She was discharged on folic acid, spironolactone, and potassium chloride. After four weeks of alcohol abstinence, she presented to the clinic with significant improvement in her symptoms. Labs at that time revealed normal urine levels of phosphate, potassium, uric acid, absent proteinuria, and no metabolic acidosis, corresponding with the treatment of Fanconi syndrome.

## Discussion

We report the case of a 33-year-old with alcohol use disorder presenting to the ED with excessive lethargy and severe dehydration secondary to repeated bouts of vomiting. Initial lab values indicated pre-renal AKI (BUN: 48, Cr: 4.05, phosphate: 9.0, anion gap: 28), for which she was volume resuscitated, resulting in improved clinical status. However, lab values following treatment were consistent with proximal tubular dysfunction suggesting possible underlying Fanconi syndrome. After an exhaustive biological workup to elucidate that the cause for her underlying disease process was negative, we attributed her syndrome to chronic alcohol abuse. 

A similar case has been reported as a letter [6] in Nephron, where a male with chronic alcohol use was found to have partial Fanconi syndrome after an AKI. Our patient was a female who presented with AKI findings that possibly masked her underlying Fanconi syndrome as she did not have the classic hypophosphatemia on presentation, which obscured a proximal tubular defect. However, with the treatment of AKI, her serum phosphate level was reduced to 2.3 mg/dl. She also had previous lab findings suggestive of chronic underlying disease compared to the partial Fanconi observed in the case described in the letter [6]. Our report presents a detailed case of a patient with atypical findings on presentation and unmasking of the more classic presentation after treatment of her AKI, which contributed to the difficulty in determining the underlying etiology.

The mechanism of alcohol use and proximal renal tubular defects is not completely known. However, some evidence suggests that since alcohol is a known mitochondrial toxin, it may interfere with the working of the Na/K ATPase pump on the basolateral membrane of the proximal tubular cells, leading to a generalized decrease in the resorptive capacity of the proximal convoluted tubule (PCT) cells [7]. Chronic alcohol use is more likely than acute intoxication to have this effect [8]. This hypothesis is reinstated in our case report as we saw a global disorder in the PCT cells of our patient, along with signs of longstanding alcohol use, such as debilitating peripheral neuropathy in her lower extremities. Our patient's underlying cause seemed to be her chronic alcohol use, as abstinence from alcohol for four weeks caused her lab abnormalities to improve.

A previous study showed that 38% of chronic alcoholics had increased fractional excretion of beta-2 microglobulin and N-acetyl B-d- glucosaminidase, which may contribute to the excretion of enzymes required by the proximal tubule and subsequent proximal tubular dysfunction [9]. This shows how alcohol may adversely affect proximal renal tubular function. Our report was limited in that we did not have beta-2 microglobulin or retinol-binding protein levels, specifically for proximal tubular dysfunction. However, our diagnosis of Fanconi was supported by classical lab findings. We could not perform a phosphatidyl ethanol (PEth) level or serum ethanol level in our patient, which would give us a more accurate measure of how much ethanol our patient had been consuming. PEth levels can be used to confirm alcohol consumption, which our patient denied at her first encounter [10]. An article published in the New England medical journal has proven that alcohol causes renal tubular damage independently [9]. 

This case highlights the importance of understanding the etiology of Fanconi syndrome in patients in order to treat it promptly. This is significant as Fanconi syndrome may present with little to no symptoms, such as in our patient. In order to avoid complications such as bone demineralization due to phosphate loss [4], it is imperative to find and treat the underlying cause of Fanconi syndrome. If not treated, it can progress to rickets in children and osteomalacia in adults, leading to a poorer quality of life [4]. Alcohol is one such cause of Fanconi syndrome that, if found, can easily be reversed with normalization of tubular function if alcohol use is stopped.

## Conclusions

In our report, we found chronic alcohol use a possible independent causative agent for Fanconi syndrome. We conclude from our case report that alcohol, owing to its wide use, could be considered a possible cause of renal tubular dysfunction with further research. It is imperative to understand the underlying etiology as we found that complete alcohol cessation can result in complete resolution of the disease process. Early diagnosis is essential in order to save time and resources. This case highlights the importance of counseling and educating patients on alcohol cessation. It is important to note that patients may underreport alcohol use, and suspicion of alcohol use contributing to renal tubular disease should be high. We require further studies to build upon this case to confirm the role of alcohol in proximal renal tubulopathies.
